# In Situ Shear Test for Revealing the Mechanical Properties of the Gravelly Slip Zone Soil

**DOI:** 10.3390/s20226531

**Published:** 2020-11-15

**Authors:** Zongxing Zou, Qi Zhang, Chengren Xiong, Huiming Tang, Lei Fan, Fang Xie, Junbiao Yan, Yinfeng Luo

**Affiliations:** 1 Three Gorges Research Center for Geo-Hazards, China University of Geosciences, Wuhan 430074, China; zouzongxing@cug.edu.cn (Z.Z.); zhangqi0416@cug.edu.cn (Q.Z.); tanghm@cug.edu.cn (H.T.); xiefangxyz@foxmail.com (F.X.); yanjunbiao@cug.edu.cn (J.Y.); luoyinfeng406@foxmail.com (Y.L.); 2 Changjiang River Scientific Research Institute, Wuhan 430074, China; fanchon1982@foxmail.com

**Keywords:** in situ shear test apparatus, mechanical property, sliding zone, shear constitutive model, Huangtupo landslide

## Abstract

Slip zone soil is usually composed of clay or silty clay; in some special geological environments, it contains gravels, which make the properties of the slip zone soil more complex. Unfortunately, in many indoor shear tests, gravels are removed to meet the demands of apparatus size, and the in situ mechanical property of the gravelly slip zone soil is rarely studied. In this study, the shear mechanical property of the gravelly slip zone soil of Huangtupo landslide in the Three Gorges Reservoir area of China was investigated by the in situ shear test. The test results show that the shear deformation process of the gravelly slip zone soil includes an elastic deformation stage, elastic–plastic deformation stage, and plastic deformation stage. Four functions were introduced to express the shear constitutive model of the gravelly slip zone soil, and the asymmetric sigmoid function was demonstrated to be the optimum one to describe the relationship of the shear stress and shear displacement with a correlation coefficient of 0.986. The comparison between the in situ test and indoor direct shear test indicates that gravels increase the strength of the slip zone soil. Therefore, the shear strength parameters of the gravelly slip zone soil obtained by the in situ test are more preferable for evaluating the stability of the landslide and designing the anti-slide structures.

## 1. Introduction

The shear mechanical properties of slip zone soil play a key role in the deformation and failure of landslides [1,2,3]. In some geological conditions, the parent rocks, such as the limestone and argillaceous limestone, near the slip zone are not completely weathered during the process of formation and evolution of the landslide, and gravels formed in the slip zone [4,5,6]. The existence of gravel significantly changes the properties of the slip zone soil [7]; therefore, it is of great significance to investigate the shear mechanical properties of slip zone soil-containing gravels for evaluating the stability and analyzing the evolution process of the landslide.

At present, the research methods of the shear mechanical properties of the slip zone soil are mainly indoor tests, including the direct shear test, ring shear test, and triaxial test in the laboratory. The direct shear test is widely used in obtaining the strength parameters of soil because of its convenience and quickness, and a lot of experience has been accumulated in engineering [8,9,10]. Landslides usually experience a process of large deformation [11]; however, the large shear displacement of the slip zone soil cannot be implemented in the direct shear test, and the ring shear test was thereby developed to study the shear mechanical properties of the slip zone soil with large displacement [12,13,14,15,16,17,18]. Compared with the direct shear test and the ring shear test, a triaxial test has the advantage of well controlling the stress condition and drainage condition, and the shear plane of soil in the triaxial test is not set artificially, which is more consistent with the actual engineering situation. Therefore, triaxial tests are often adopted to study the mechanical properties of slip zone soil [19,20]. However, the soil is usually remolded in the above laboratory tests, which destroy its original structure. Moreover, the sizes of the test samples are limited by the test equipment, which fails to study the mechanical properties of the slip zone soil containing gravels. Consequently, the laboratory test results are usually inconsistent with the actual mechanical properties of the gravelly slip zone soil.

With the improvement of large-scale engineering and conditions, in situ tests are becoming more and more popular [21], as they can overcome the structural disturbance of the slip zone soil and realize the test of soil containing gravel. However, the in situ shear tests are rarely implemented to study the shear mechanical properties of the gravelly slip zone soil. Therefore, it is significant to carry out the in situ shear test for the gravelly slip zone soil to reveal their in situ shear mechanical properties.

The objective of the study is to investigate the in situ shear mechanical properties using an in situ shear test apparatus. In this study, an in situ direct shear apparatus was designed and applied to investigate the shear mechanical properties of the gravelly slip zone soil of the Huangtupo landslide in the Three Gorges Reservoir area. On the basis of the tests, constitutive models of the gravelly slip zone soil were proposed to describe the relationship of the shear stress and shear displacement. The in situ test was further compared with the laboratory test, and the differences of the obtained mechanical properties of the slip zone soil by two methods were analyzed.

## 2. Materials and Methods

### 2.1. Site Description

**Figure 1:**
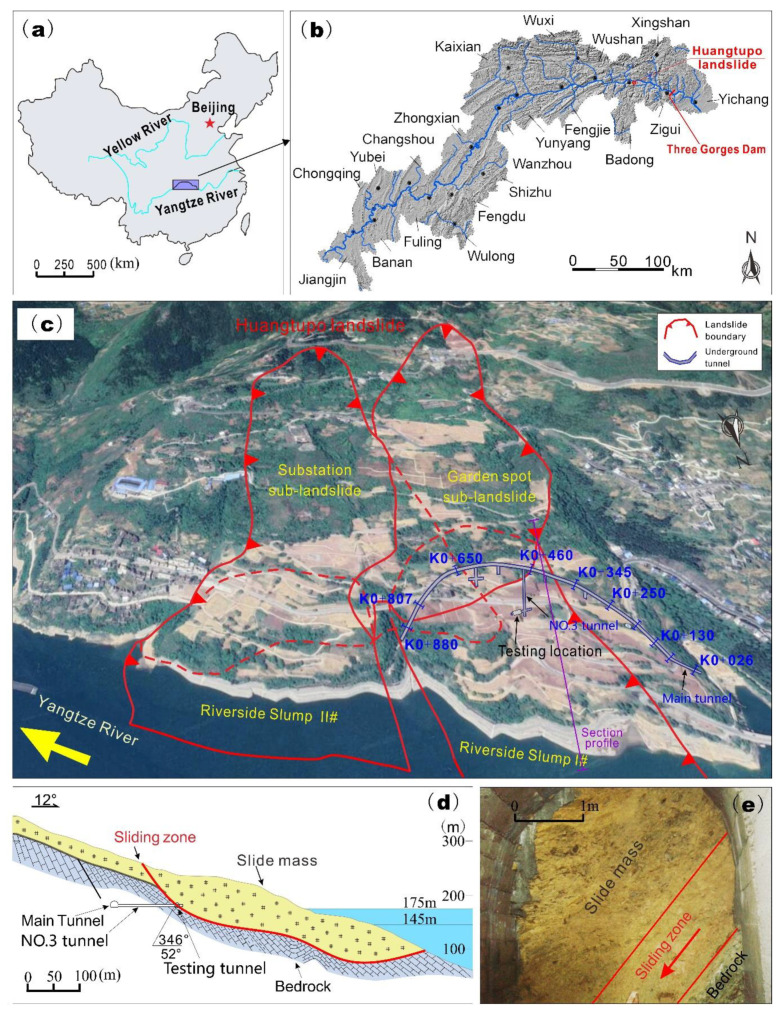
(**a**,**b**) show the location of the Huangtupo landslide; (**c**,**d**) are the map and profile section, respectively, of the Huangtupo landslide; (**e**) is the face of the testing tunnel where the in situ shear tests were conducted.

To investigate the mechanical property of the gravelly slip zone soil, a group of in situ shear tests were conducted in the sliding zone of the Huangtupo landslide, which is volumetrically the largest reservoir landslide in the Three Gorges Reservoir area, having a volume of approximately 70 million m^3^ [22,23]. The Huangtupo landslide is located in Badong County of Hubei Province in China (Figure 1a) on the south bank of the Yangtze River about 72 km upstream from the Three Gorges dam (Figure 1b). It has been quite active since reservoir impoundment, and it posed a great threat to a community with more than 16,000 people [24]. For this reason, a billion RMB was invested to relocate the residents from the Huangtupo landslide.

The Huangtupo landslide consists of four sub-landslides (Figure 1c), including the Riverside Slump I# (northwest part), Garden spot sub-landslide (southwest part), Riverside Slump II# (northeast part), and Substation sub-landslide (southeast part) [25]. The stability of this large landslide, especially Riverside Slump I#, is of great concern regarding the safety of the Yangtze River main channel. Therefore, a large in situ experimental station was constructed for monitoring, evaluating, and researching this crucial landslide. This experimental station consists of underground tunnels (Figure 1) and a series of multi-filed monitoring systems. The tunnels include a 908-m-long main tunnel, five branch tunnels with lengths of 5–145 m, and two testing tunnels of about 10-m length [26]. The tunnel groups not only reveal the spatial structure of the landslide, but also expose the sliding zone, which can provide powerful site conditions for the in situ shear test of the slip zone soil.

The Riverside Slump I# has an average thickness of 69.4 m, an average N–S length of 770 m, a width of 475 m in the W–E direction, and a volume of approximately 23 million m^3^. The slide mass of the Riverside Slump I# mainly contains cataclastic rock, rock block, and rock fragments with clay [27]. The sliding zone is composed of silty clay with gravels and has a thickness of 0.5–1 m. The slide bed mainly consists of argillaceous limestone. In this study, in situ shear tests were conducted in the testing tunnel that connects to the No.3 branch tunnel (Figure 1c,d). The moisture content and density of the undisturbed slip zone soil sample taken from the testing site were tested (Figure 1e). The natural density, moisture content, and dry density of the slip zone soil are 2.31–2.43 g/cm^3^, 12.9–18.1%, 2.09 g/cm^3^, respectively. The slip zone soil is typically composed of fine-grained soil (silty clay) with a significant fraction of coarse-grained particles (gravels), constituting approximately 20–50 wt % (weight percent) of the material [6].

### 2.2. Principle of In Situ Shear Test

**Figure 2:**
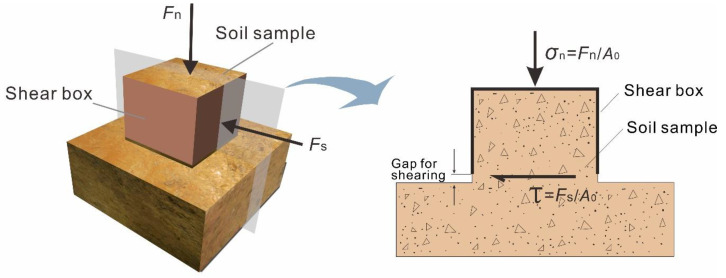
Diagram showing the in situ shear test.

The principle of the in situ shear test is basically the same as the indoor shear test (see Section 2.5 for details). The obvious advantage of the in situ test is that the samples in the in situ shear test can avoid disturbance and keep the original structure of the slip zone soil. Under the condition of keeping the soil in its original state, a normal load (*F*_n_) is firstly applied in the normal direction to generate the normal stress. After the normal deformation induced by the normal load is relatively stable, a thrust (*F*_s_) is to be slowly applied to produce shear stress on the preset shear surface (Figure 2). When the sample steps into the failure state, an ultimate steady shear stress is acquired and is named the shear strength (*τ*_f_). Various ultimate shear strengths are obtained by conducting a series of in situ shear tests with different normal stresses. Taking the normal stress as an abscissa value and the shear strength as an ordinate value, the slope and intercept of the straight line can be obtained by linearly fitting the data with the least square method. The intercept value obtained represents the value of the cohesion; the value of the arctangent of the slope is the value of the internal friction angle of the slip zone soil. The equation of *τ*_f_ -*σ*_n_ can be expressed as follows:

(1) $$\tau_{f}=\sigma_{n}\tan\varphi +c$$

where *τ*_f_ is the shear strength of the slip zone soil; *σ*_n_ is the normal stress; and *φ* and *c* are the internal friction angle and the cohesion of the slip zone soil, respectively.

### 2.3. In Situ Shear Test Apparatus

**Figure 3:**
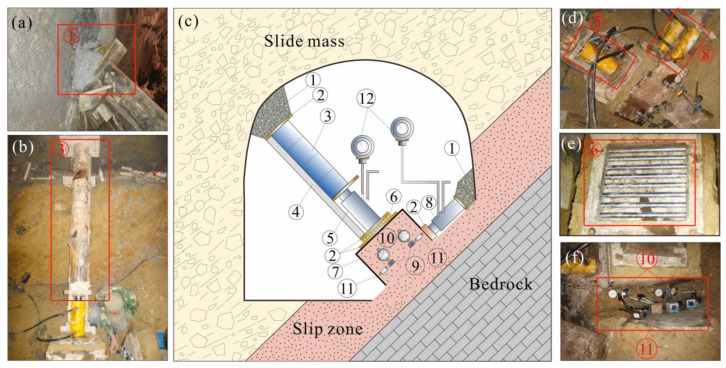
The composition of the in situ shear test apparatus. ① Reaction wall (as shown in (**a**)); ② steel backing plate; ③ transfer column (as shown in (**b**)); ④ guiding part; ⑤ the normal hydraulic jack (as shown in (**d**)); ⑥ steel rollers (as shown in (**e**)); ⑦ shear box; ⑧ tangentially arranged hydraulic jacks; ⑨ steel stick; ⑩ normal displacement gauge (as shown in (**f**)); ⑪ horizontal displacement gauge (as shown in (**f**)); ⑫ pressure gauge.

The in situ shear tests of the slip zone soil are carried out in the field and usually in remote mountainous areas, so the in situ shear test apparatus needs to be convenient to carry. The shear test apparatus designed in this experiment has the advantage of being detachable, portable, and easy to install. This in situ shear apparatus is composed of loading devices, force transmitting devices, reaction walls, a shear box, and measurement instrumentations (Figure 3).

The loading devices include one hydraulic jack providing force on the normal direction of the sample (⑤ in Figure 3) and two hydraulic jacks providing force on the shearing direction of the sample (⑧ in Figure 3). The normal hydraulic jack that provides normal force on the sample can produce a maximum working force of 500 kN, and it has an effective action area of 80 cm^2^. To prevent the shear box from deflection, two tangentially arranged hydraulic jacks are adopted to load the thrust in the shear direction. Both the tangentially arranged hydraulic jacks can produce a maximum working pressure of 300 kN and have an effective action area of 60 cm^2^. These hydraulic jacks have a 100-mm stroke, implying that the maximum shear and normal displacement of the jacks are 100 mm.

Force-transmitting devices transmit the jack pressure to the reaction wall on the one hand and transmit the jack pressure to the sample smoothly on the other hand. The force-transmitting devices include a steel backing plate, transfer column, guiding part, and steel rollers (②, ③, ④, ⑥ in Figure 3, respectively). The transfer column is assembled by a series of short steel columns with a length of 40 cm to meet the requirement of different testing tunnel sizes. In order to prevent the force transfer column from deflexion as the result of the shear movement of the sample during the shear test, two steel backing plates and a set of steel rollers are set between the normal hydraulic jack and the slip zone soil sample. The size of the steel backing plates is 40 cm × 40 cm, and the steel rollers are arranged to form a size of 30 cm × 30 cm. Force-transmitting devices should have a large stiffness to reduce the deformation of the devices during the loading process, so as to reduce the deformation measurement error of the sample.

Measurement instrumentations include two pressure gauges (⑫ in Figure 3) and eight displacement gauges (⑩ and ⑪ in Figure 3). The pressure gauges are adopted to measure the pressure of the normal and tangentially arranged hydraulic jacks. Displacement gauges are employed to measure the shearing displacement and normal displacement during the shearing process. The precision of the pressure gauge is 0.01 MPa. The precision of the shear and normal displacement gauges are 0.01 mm and 0.001 mm, respectively.

The inner size of the shear box is 50 cm in length, 50 cm in width, and 40 cm in height (⑦ in Figure 3). There are four observation holes in the side of the shear box, and steel sticks (⑨ in Figure 3) are inserted via these holes, providing access for measuring the displacement of the slip zone soil sample.

The reaction wall is casted in site to fit the posture of the transfer column and the shear box, ensuring that the force produced by the hydraulic jacks is smoothly transmitted to the tunnel wall. The reaction wall (① in Figure 3) was casted with fast hardening concrete in this experiment to shorten the forming time of the building reaction wall.

### 2.4. In Situ Shear Test Process

**Figure 4:**
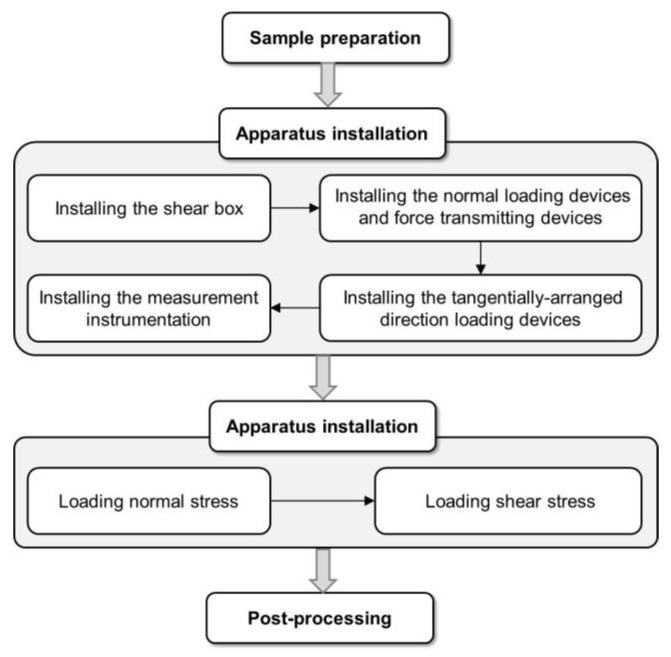
Flow chart for the in situ shear test of slip zone soil.

The general process for the in situ shear test of the slip zone soil is shown in Figure 4.

#### 2.4.1. Sample Preparation

**Figure 5:**
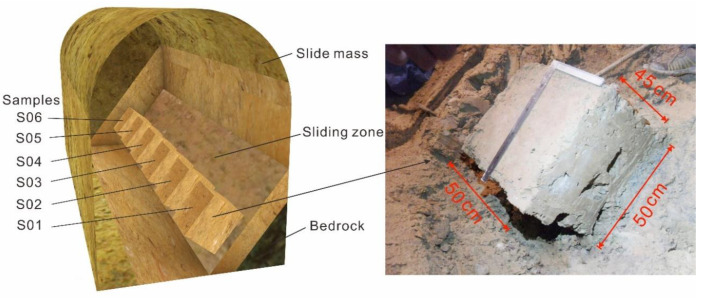
Prepared samples for the in situ shear test in the No.3 testing tunnel in the Huangtupo landslide.

Sliding zones are exposed by the tunnels in many places of the Huangtupo landslide field experimental station, and the most observable one is in the east of No.3 branch tunnel; therefore, this site was selected for the in situ shear test. The bedrock exposed at this location dips 52° toward 346° (Figure 1d). The shear test samples were made in the sliding zone. The shear direction of the sample is consistent with the bedrock tendency. The sample is a cuboid, having a size of 50 cm × 50 cm × 45 cm (length × width × height), as shown in Figure 5. Gravels with larger diameter on the surface of the sample are removed to make the surface of the sample smooth, and the corresponding dents were treated with slip zone soil, so that the samples can better contact with the apparatus. Six samples were prepared and marked as S01–S06 from the outside to the inside of the testing tunnel (Figure 5).

#### 2.4.2. Apparatus Installation

The installation procedures of the in situ shear test apparatus can be divided into the following steps:

The first step was to install the shear box. After the sample was made, we slowly applied pressure and pressed the shear box into the sample until the distance between the lower edges of the shear box and the bottom of the sample was about 5 cm. This 5-cm thick soil was preserved for the shearing space (Figure 2), which is greater than the maximum gravel size in the sliding zone. The gap between the shear box and ground was filled with slip zone soil. Then, we leveled the soil sample at the top of the shear box to facilitate the installation of subsequent devices.

The second step was to install the normal loading devices and force-transmitting devices. A 40 cm × 40 cm steel plate was placed on top of the sample. Then, the steel rollers (Figure 3e) were placed on the steel plate to prevent the eccentricity of a normal load in the shearing process. We put another steel plate on the steel rollers and installed the hydraulic jack. Then, we put the steel plate on the back of the hydraulic jack and installed the short steel columns behind the steel plate until the top of the steel column reached the chamber wall, and finally, we cast the reaction wall.

The third step was to install the tangentially arranged direction loading devices. A steel plate was placed on the side of the shear box as the force transfer plate of the tangential jack, and then the hydraulic jack, transfer column, and reaction wall were installed successively. When the location of the sample was close to the testing tunnel wall, the transfer column could be omitted, and only reaction walls were retained.

The fourth step was to install the measurement instrumentation. There were two observation holes on both sides of the shear box, 10 cm away from the bottom. Steel sticks were inserted into the soil sample through the observation holes, and normal and horizontal displacement gauges were installed on the steel sticks.

**Figure 6:**
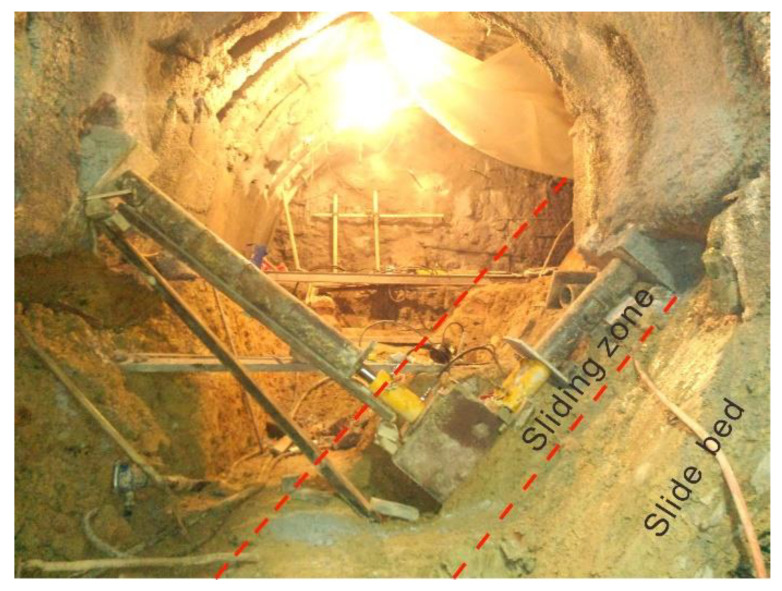
Full view of the installed apparatus of the in situ shear test in the testing tunnel of the Huangtupo landslide.

The full view of the installed apparatus for the in situ shear test in the testing tunnel of the Huangtupo landslide is shown in Figure 6.

#### 2.4.3. Stress Loading

The maximum normal stress is estimated according to the thickness of the overburden layer of the sliding mass. Then, the normal pressure applied on each soil sample is determined. During the shearing process, the normal pressure of the test sample was kept stable by continuously adding pressure to the hydraulic jack. The normal stress was loaded step by step through three to four levels. The meter reading should be conducted every three minutes in each level. When the difference between the two consecutive readings of the normal displacement was less than 0.01 mm, indicating that the deformation is relatively stable, the next level load was applied. After the last level of load was applied, the normal deformation should be measured and recorded at an interval of 5 min. When the accumulated normal displacement of two consecutive 15 min was less than 0.01 mm, the normal deformation was considered to be stable, and then the shear load was applied.

According to the estimated maximum shear strength, the shear load was applied step by step through 8–12 levels, and the load of each level was applied every 10 min. When the shear displacement increased sharply or reached 1/10 of the sample size (50 cm), the shearing surface cannot bear more stress, it was considered to be damaged, and the test was terminated. The readings of all gauges were recorded immediately before and after each load was applied.

#### 2.4.4. Post-Processing

The normal stress (*σ*_n_) and shear stress (*τ*) can be calculated by following equations:

(2) $$\sigma =\frac{F_{n}}{A_{0}}$$

(3) $$\tau =\frac{F_{s}}{A_{0}}$$

where *F*_n_ and *F*_s_ are the normal load and shear load acting on the shear plane, respectively; *A*_0_ is the area of the shear plane of the slip zone soil sample.

In this test, the pressure readings of the final normal hydraulic jack (*P*_n_) are 2.1, 2.5, 4.4, 5.9, 7.9, and 9.9 MPa, respectively. The cross-sectional area of a normal jack cylinder (*A*_1_) is 80 cm^2^. The size of the shear box is 50 cm × 50 cm × 40 cm (length × width × height), so the normal stress area of the slip zone soil sample (*A*_0_) is 2500 cm^2^; consequently, the normal stress of the sample can be estimated by the following formula:

(4) $$\sigma =\frac{F_{n}}{A_{0}}=\frac{P_{n}A_{1}}{A_{0}}=0.032P_{n}$$

Therefore, the final normal stresses of six samples are estimated as 0.067, 0.080, 0.141, 0.189, 0.253, and 0.317 MPa, respectively.

Two tangentially arranged direction hydraulic jacks were installed for providing the shear load. The cross-sectional area of each jack cylinder (*A*_2_) was 60 cm^2^. The two jacks were pressurized by the same hydraulic system, and their pressures were displayed by the same pressure gauge. During the shearing process, the shear area decreased with the increase of shear displacement. Since the total shear displacement was relatively small compared with the size of the slip zone soil sample. Therefore, the area of the shear plane was estimated to be the cross-sectional area of the sample *A*_0_ (2500 cm^2^). The shear stress *τ* can be calculated by the following formula:

(5) $$\tau =\frac{F_{s}}{A_{0}}=\frac{2P_{s}A_{2}}{A_{0}}=0.048P_{s}$$

**Figure 7:**
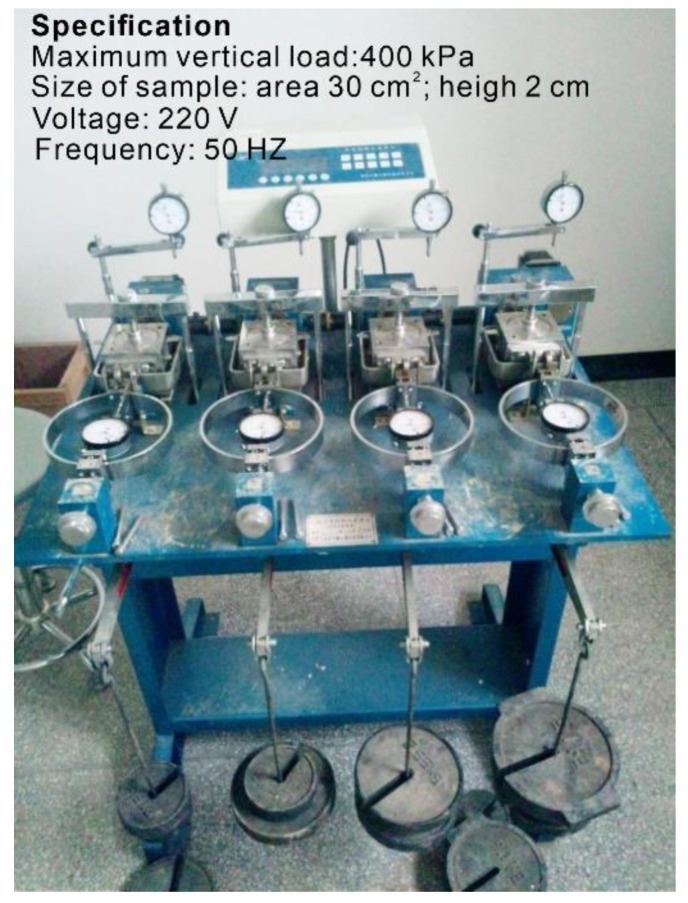
Indoor direct shear test apparatus.

The strain-controlled direct shear apparatus (Figure 7) was adopted for this indoor direct shear test. The remolded soil sample had a diameter of 61.8 mm and a height of 20 mm. The gap between the upper and lower shear boxes is about 0.1 mm. It was sheared at a shear rate of 0.8 mm/min. Four samples were sheared under normal pressures of 100, 200, 300, and 400 kPa, respectively.

### 2.5. Indoor Direct Shear Test

In order to compare with the shear strength parameters (*c*, *φ*) of the slip zone soil obtained by the in situ shear test, a series of the indoor direct shear test of the remolded slip zone soil was carried out. The location of the soil samples taken in the laboratory test was the same as that of the in situ test. The large particles in soil samples were removed during the test. To meet the requirements of the test instrument for the particle size of soil, a 2-mm-sieving was used to sieve the slip zone soil. Then, a group of screened soil samples with moisture content of 13.63% was prepared by the dynamic compaction method. The dry density and target density of the prepared sample are 2.07 g/cm^3^ and 2.37 g/cm^3^, respectively.

## 3. Results

### 3.1. Deformation of the Sample during Shear Test

**Figure 8:**
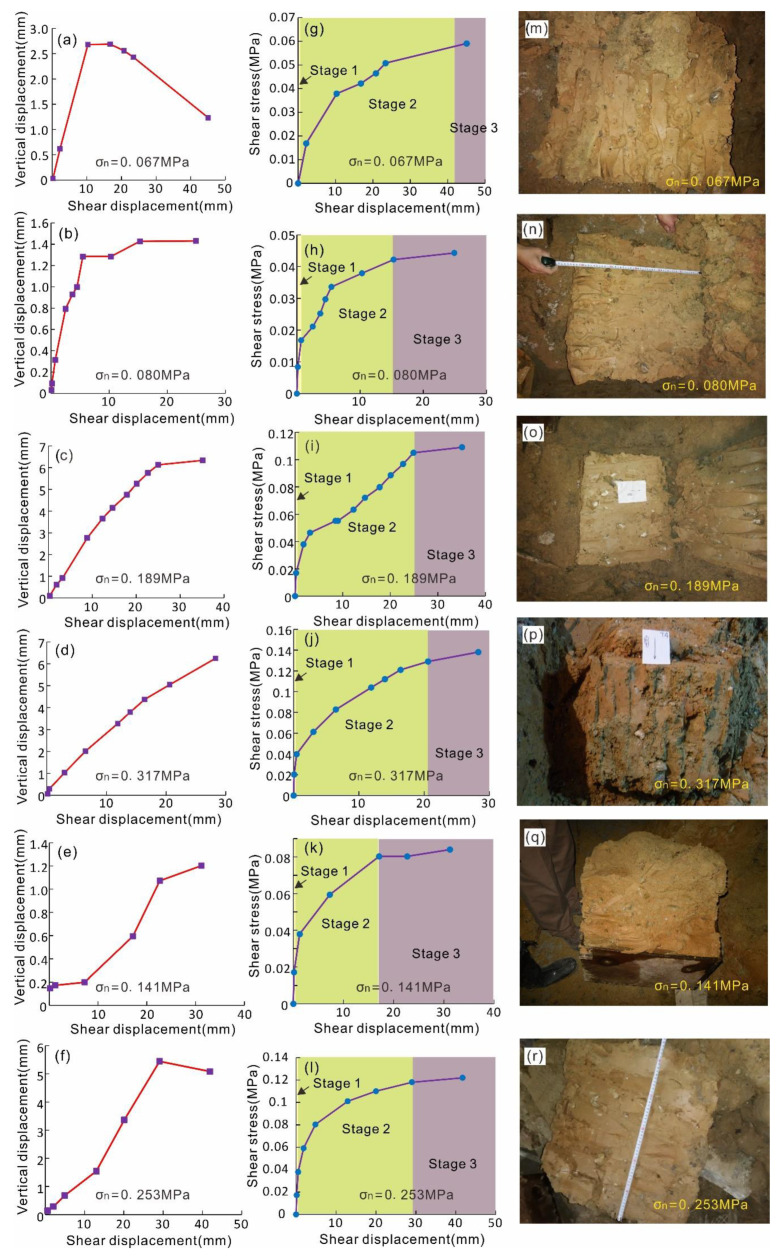
(**a**–**f**) Shear stress with the increase of shear displacement under various normal stress conditions. (**g**–**l**) Normal displacement of the samples with the increase of the shear displacement under various normal stress conditions. (**m**–**r**) Shear plane surface of the samples after the shear test.

The test results of six samples under different normal stresses are shown in Figure 8.

It can be seen from the normal displacement–shear displacement curves (*u*_n_–*u*_s_ curves) in Figure 8a that during the shearing process of sample S01, the normal displacement of the soil sample increases firstly and then decreases; that is, the soil sample rebounds in the later stage. This may be related to the rotation of the larger gravels near the shear plane during the shearing process (Figure 8m). During the shearing process, the normal displacement of samples S02 and S03 increases gradually in the early stage, and then, it tends to be stable (Figure 8b,c). The normal displacement of sample S04 increases steadily with the increase of shear displacement, and the normal displacement does not converge obviously (Figure 8d). In the process of the shear test, the normal displacement of sample S05 increases slowly; then, it accelerates and finally slows down, and the total displacement is very small, about 1.2 mm (Figure 8e). The normal displacement of sample S06 increases slowly at first; then, it increases rapidly, and finally, it decreases again (Figure 8f), which is similar to sample S01. The rebound in the normal direction may be attributed to the movement mode of large gravels on the shear plane (Figure 8r).

Figure 8 shows that the shape of shear stress–shear displacement curves (*τ*–*u*_s_ curves) are different. Since the in situ slip zone soil is inhomogeneous, the structure and composition of the sample are not exactly the same. The slope of the *τ*–*u*_s_ curve of S01, S02, and S03 decreases, increases, and then decreases again with the increase of shear displacement (Figure 8g,h,i). While in the samples S04, S05, and S06, the slope of the curves shows a gradual decrease and the curves eventually tend to be horizontal (Figure 8j,k,l). The inhomogeneity of the material makes the test data irregular. For example, the normal stress of the S01 sample is 0.067 MPa, which is smaller than that of sample S02 (0.080 MPa), but its shear strength is larger than that of sample S02.

In general, the *τ*–*u*_s_ curves exhibit that with the increase of shear displacement, the slope of each curve gradually decreases, and curves finally tend to be stable. According to the deformation and failure feature of the slip zone soil, *τ*–*u*_s_ can be divided into three stages, and it is described as follows:

(1) Elastic deformation stage: In this stage, the sample just begins to be stressed and deformed, and the shear stress–shear displacement curve is approximately linear. Tiny deformation is generated at the bottom of the sample, and no complete shear plane is formed.

(2) Elastic–plastic deformation stage: In this stage, the internal fracture of the sample begins to occur, but the propagation speed of the fracture is relatively slow. Some irregular change of the *τ*–*u*_s_ curve may appear in this stage (such as S01, S02, S03). The slope of the curve of *τ*–*u*_s_ decreases gradually.

(3) Plastic deformation stage: With the continuous increase of shear displacement, the shear plane has basically formed in this stage; the plastic flow occurs, and the shear stress tends to be stable. 

The characteristic of the gravelly slip zone soil is different from that of the slip zone soil without gravels. Previous study shows that the slip zone soil without gravels exhibits a phenomenon of strain softening. The typical full process of shear deformation and failure of the slip zone soil can be divided into five stages, including the pore shear compaction stage, the elastic deformation stage, the plastic hardening stage, the strain softening stage, and the residual strength stage [2].

### 3.2. Shear Strength of the Slip Zone Soil by In Situ Shear Test 

**Figure 9:**
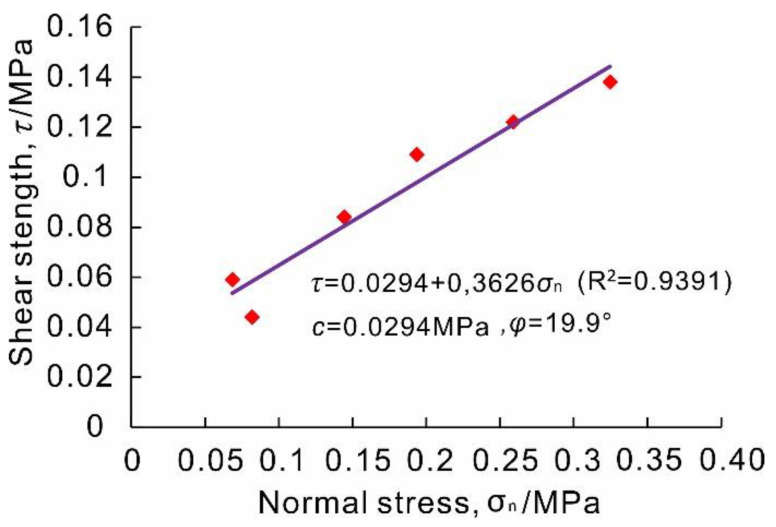
Shear strength of the slip zone soil of the Riverside Slump I# of the Huangtupo landslide obtained by in situ shear tests.

According to the curve of shear stress–shear displacement (Figure 8g–l), the shear strength values (the maximum value in the curve) under various normal pressures are determined. Under the normal stresses of 0.067, 0.080, 0.189, 0.317, 0.141, and 0.253 MPa, the shear strengths are 0.059, 0.044, 0.109, 0.138, 0.084, and 0.122 MPa, respectively. Furthermore, the relationship curve of *τ*–*σ*_n_ can be drawn (Figure 9), and the linear fitting equation is obtained according to the least square method:

(6) $$\tau =0.363\sigma_{n}+0.0294\;(R^{2}=0.9239)$$

Consequently, the shear strength parameters of slip zone soil of the Riverside Slump I# of the Huangtupo landslide can be obtained. The cohesion is 29.4 kPa, and the internal friction angle is 19.9°, which is the arctangent of 0.3626.

### 3.3. Shear Constitutive Model of Slip Zone Soil with Gravels

The constitutive model (quantitative relationship between stress and displacement) of slip zone soil is an important basis for analyzing the mechanical behavior of landslides. Currently, many stress–strain constitutive models have been developed based on laboratory tests; however, few stress–displacement constitutive models [2] and few shear constitutive models based on in situ tests have been developed. It can be seen from the shear stress–shear displacement curves (Figure 8) that the initial curve is steep in the elastic stage; with the increase of shear displacement, the slope of the curve gradually decreases, and finally, the shear stress value tends to be stable. Considering the above characteristics of the curves, four kinds of equations, namely asymmetric sigmoid function, symmetric sigmoid function, exponential function, and power function, are introduced to describe the shear mechanical behavior of slip zone soil, and the shear stress–shear displacement constitutive model is thereby established.

The asymmetric sigmoid function is expressed as follows:

(7) $$\tau =A-\frac{A}{1+{\left(\frac{u_{s}}{u_{0}}\right)}^{p}}$$

The symmetric sigmoid function is expressed as follows:

(8) $$\tau =\frac{A}{1{+e}^{-k\left(u_{s}-u_{0}\right)}}$$

The exponential function is expressed as follows:

(9) $$\tau =A-a\cdot e^{-u_{s}/u_{0}}$$

The power function is expressed as follows:

(10) $$\tau =a\cdot u_{s}{}^{p}$$

*A*, *u*_0_, *p*, *k*, *a* are the relative parameters of the above equations. Each equation has two to three parameters.

**Figure 10:**
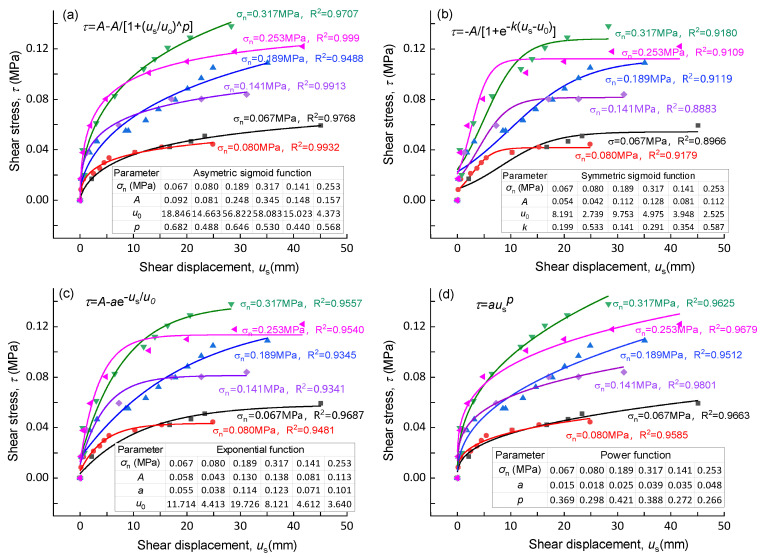
Fitting curves with four functions for based on in situ shear test data. (**a**) Asymmetric sigmoid function; (**b**) symmetric sigmoid function; (**c**) exponential function; (**d**) power function.

**Figure 11:**
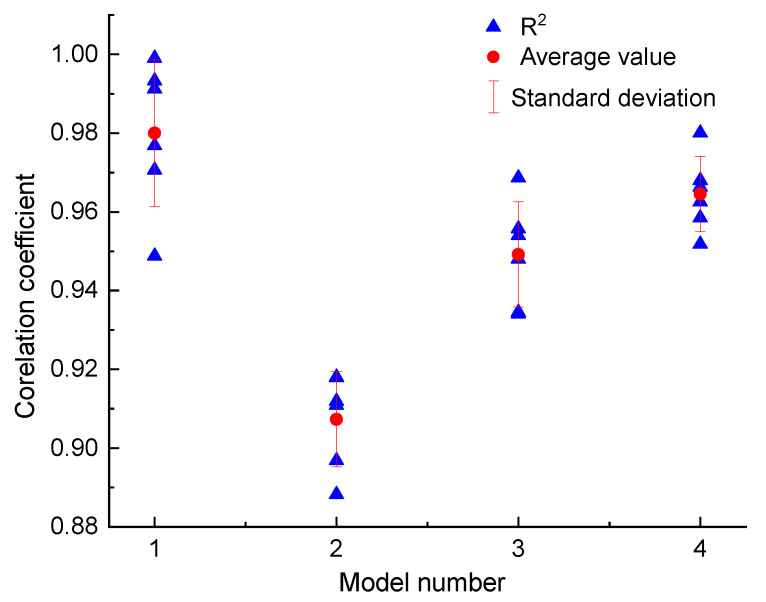
Correlation coefficients for different functions. 1 Asymmetric sigmoid function; 2 symmetric sigmoid function; 3 exponential function; 4 power function.

Figure 10 shows the fitting results of in situ shear test data of the slip zone soil of the Riverside Slump I# of the Huangtupo landslide using the above formulas. On the whole, the test data are well fitted by the equations mentioned above, as demonstrated by the correlation coefficient of each group of curves with values above 0.9 (Figure 11). Among the four formulas, the asymmetric sigmoid function shows the best fitting, with an average correlation coefficient of 0.980, followed by a power function, exponential function, and symmetric sigmoid function, with average correlation coefficients 0.963, 0.948, and 0.907, respectively (Figure 11).

As can be seen from Figure 10b, most of the fitting curves of the symmetric S-curve do not pass through the initial point and maximum point, and the fitting curve changes greatly from the actual point position when the normal stress *σ*_n_ = 0.253 MPa. Figure 10c shows that although the fitting degree of the curve is high, the change from the steep part to the gentle part is too sudden to express the elastic–plastic stage. Figure 10d shows that data are well fitted, but it cannot reflect the feature of shear stress tending to be stable in the plastic stage, and the curve cannot converge; therefore, it cannot be adopted. The curves in Figure 10a fit the characteristics of each stage well. 

To sum up, the asymmetric sigmoid function not only has a high fitting degree with the test results, but it also defines the relationship between shear stress and shear displacement in the process of the direct shear test, which can be applied to analyze the change of shear stress with shear displacement in each stage of the direct shear test. The asymmetric sigmoid function is the best for constructing the shear constitutive model of the gravelly slip zone soil. The parameters in this proposed shear constitutive model have clear physical meanings. The value of *A* in Equation (7) represents the yield strength of slip zone soil, and parameters *a* and *u*_0_ jointly control the shape of the constitutive curve. The above three parameters change with the normal stress value of the slip zone soil.

The presented model describes the stress–displacement relations. It is significant for landslide stability analysis, because the shear stress varies as the slip surface displaces. Utilizing the shear–displacement model, the dynamic stability of the landslide can be evaluated [2]. Moreover, the shear strength parameters can be derived from the shear constitutive model; therefore, this shear constitutive model is of great use in practice.

## 4. Discussion

**Figure 12:**
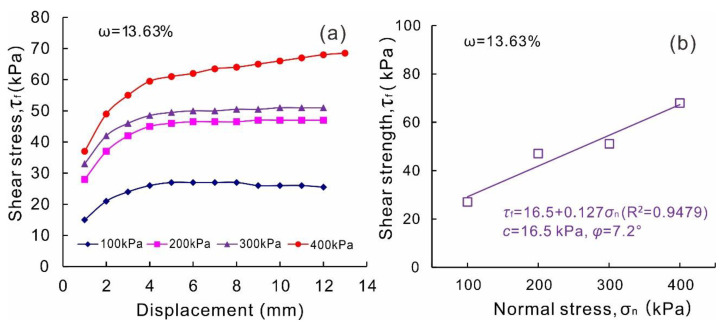
Indoor direct shear test results of the 2-mm-sieved slip zone soil of the Riverside Slump I# of the Huangtupo landslide with water content of 13.63%. (**a**) Shear stress vs. displacement under various normal stress; (**b**) shear strength vs. normal stress.

The indoor direct shear test results are shown in Figure 12. The shear strength parameters measured by the indoor direct shear test are 16.5 kPa for cohesion and 7.2° for internal friction angle. Compared with the results of the in situ direct shear test and laboratory test, it can be found that the cohesion decreases by 43.9%, and the internal friction angle decreases by 63.8%. The strength of the slip zone soil is underestimated by the indoor direct shear test, which may be attributed to the following reasons. Firstly, the structure of the slip zone soil was disturbed in the remolded soil sample. More importantly, gravels in the slip zone soil were removed due to the size limitation of the indoor direct shear test apparatus. In the in situ test, the content of coarse particles in the soil sample is higher, and the interaction and friction between the particles are more severe than those in the indoor direct shear tests. So, the internal friction angle of the soil sample is greatly higher than that of the indoor direct shear test. It further reveals the important role of the gravels played in the slip zone soil strength. Since the shear strength parameters are the basic data for evaluating the stability of the landslide and designing the anti-slide structures for landslides, the shear strength parameters obtained by the in situ test are more preferable.

The in situ shear test has many advantages such as preserving the original structure of the slip zone soil and allowing large shear displacement; however, it is time-consuming and expensive. Nevertheless, the in situ shear test is affordable and preferable in many important projects.

## 5. Conclusions

Utilizing the large-scale field comprehensive landslide experimental base in the Three Gorges Reservoir area, a group of in situ shear tests of the gravelly slip zone soil of the Riverside Slump I# of the Huangtupo landslide were conducted by using the designed portable apparatus. The shear mechanical properties of the gravelly slip zone soil were analyzed. The following conclusions are reached:

(1) In the later stage of the shear test, rebound may occur along the normal direction of the sample. The rebound phenomenon is related to the rotation of the gravels on the shear plane. 

(2) The shearing process of the gravelly slip zone soil of the Huangtupo landslide revealed by the in situ shear test can be divided into three stages including an elastic deformation stage, elastic–plastic deformation stage, and plastic deformation stage.

(3) Four types of functions were introduced to describe the relationship of the shear stress and shear displacement of gravelly slip zone soil. The asymmetric sigmoid function is demonstrated to be the optimal one and the parameters of the function have clear physical meaning.

(4) The shear strength of the slip zone soil obtained by the indoor direct shear test is underestimated compared with the in situ shear test result. The gravels have critical influence on the mechanical property of the slip zone soil, and the in situ shear test is preferable to obtain the shear strength of the gravelly slip zone soil for reliable landslide stability evaluation and anti-slide structure design.
